# Metabolic Imaging of Deep Brain Stimulation in Meige Syndrome

**DOI:** 10.3389/fnagi.2022.848100

**Published:** 2022-03-17

**Authors:** Jiayu Liu, Lei Li, Yuan Li, Qian Wang, Ruen Liu, Hu Ding

**Affiliations:** ^1^Department of Neurosurgery, Peking University People’s Hospital, Beijing, China; ^2^Department of Nuclear Medicine, Zhuhai People’s Hospital (Zhuhai Hospital Affiliated With Jinan University), Zhuhai, China; ^3^Department of Nuclear Medicine, Peking University People’s Hospital, Beijing, China

**Keywords:** positron emission tomography, meige syndrome, deep brain stimulation, subthalamic nucleus, glucose metabolism

## Abstract

**Objectives:**

The subthalamic nucleus (STN) has been shown to be a safe and effective deep brain stimulation (DBS) surgical target for the treatment of Meige syndrome. The aim of this study was to compare changes in brain metabolism before and 6 months after STN-DBS surgery.

**Methods:**

Twenty-five patients with primary Meige syndrome underwent motor function assessment, including the Burke–Fahn–Marsden Dystonia Rating Scale movement (BFMDRS-M) and disability subscale (BFMDRS-D) and positron emission tomography with an 18[F]-fluorodeoxyglucose scan before and 6 months after STN-DBS surgery. For the voxelwise metabolic change assessment, the *p*-value was controlled for multiple comparisons using the familywise error rate.

**Results:**

There was a significant decrease in BFMDRS-M scores 6 months after STN-DBS, from 10.02 ± 3.99 to 4.00 ± 2.69 (*p* < 0.001). The BFMDRS-D scores also decreased significantly from 4.52 ± 2.90 to 0.64 ± 1.29 (*p* < 0.001). In the left hemisphere, hypermetabolism was found in the occipital lobe, superior parietal gyrus, postcentral gyrus and thalamus. In the right hemisphere, hypermetabolism was found in the lentiform nucleus, precuneus and precentral gyrus in patients with Meige syndrome receiving DBS. In addition, the bilateral inferior temporal gyrus and middle frontal gyrus exhibited glucose hypermetabolism.

**Conclusion:**

Our findings indicate that STN-DBS has a significant effect on metabolic level in the brain, which may be an important mechanism for the treatment of Meige syndrome using STN-DBS.

## Introduction

Meige syndrome is an adult-onset dystonia, primarily involving cranial and neck muscles and characterized by blepharospasm and dystonia of the mouth, jaw and neck. Blepharospasm is blinking or other eyelid movements (like twitching, that you can’t control), which is an important feature of Meige syndrome. Defazio et al. (2006) estimated the crude prevalence rate for segmental dystonia as 59 per million. Meige syndrome occurs most frequently between the ages of 40 and 70 and is two to three times more common in women than in men. Most patients experience an insidious onset with gradual progression. Continuous abnormal facial muscle contractions can lead to visual impairment, even functional blindness, as well as dysfunction of chewing, swallowing, and speech, which can seriously affect the patient’s daily life (Faulstich et al., 1985).

The pathogenesis of Meige syndrome has not been fully elucidated. Dystonia is generally considered to be related to the basal ganglia-thalamocortical motor circuit (Colosimo et al., 2010; Neychev et al., 2011). In our previous voxel-based morphology study, we found that the precuneus is involved in the development of Meige syndrome (Liu et al., 2020). However, this study did not find abnormalities in the basal ganglia region in patients with Meige syndrome, which may be related to basal ganglia dysfunction rather than pathological/anatomical abnormalities. Understanding the patterns of brain metabolism in Meige syndrome may help to target the underlying brain functional abnormalities, understand the pathophysiology of the disease, and conduct surgical treatment. Metabolic brain imaging using 18F-FDG PET provides a means of quantifying changes in spatially distributed neural systems in neurological diseases (Zhang et al., 2013). Our FDG PET study compared patients with Meige syndrome to healthy control (HC) subjects and found that hypometabolism in the globus pallidus and thalamus may indicate basal ganglia-thalamocortical motor circuit abnormalities as a pathogenic mechanism of Meige syndrome (Liu et al., 2021b). This provides a possible explanation for the efficacy of deep brain stimulation (DBS) in improving symptoms.

Deep brain stimulation can be used to treat Meige syndrome by implanting stimulating electrodes into the basal ganglia nuclei and continuously releasing electrical signals through a pulse generator to inhibit abnormal discharge and regulate the basal ganglia motor circuits. The globus pallidus internus (GPi) is the most widely targeted region in deep brain stimulation in Meige syndrome (Tian et al., 2019; Hao et al., 2020). Our previous study also demonstrated the safety and efficacy of subthalamic nucleus (STN) DBS (Ouyang et al., 2021), revealing that patients with Meige syndrome had similar improvements in motor function, quality of life and sleep after either GPi-DBS or STN-DBS (Liu et al., 2021a).

However, the effects of DBS on brain function are not entirely understood in Meige syndrome. PET studies can be applied to monitor the DBS-induced metabolic response. Voxel-based statistical techniques, such as those found in SPM software, have been widely applied in functional neuroimaging studies, including PET. We demonstrated that there was a pattern of abnormal metabolic regions and pathophysiological networks in Meige syndrome patients. In this study, we hypothesized that metabolism would be modified after STN-DBS.

## Materials and Methods

### Participants

Data from 25 right-handed patients with primary Meige syndrome undergoing STN-DBS were collected between September 2017 and January 2021 at the Department of Neurosurgery, Peking University People’s Hospital. All patients were told to stop oral medication before surgery. These patients underwent PET-FDG before and 6 months after surgery. The diagnostic criteria were primarily based on the presence of blepharospasm, oromandibular dystonia and cervical dystonia, increased blink rates and other symptoms (Faulstich et al., 1985). Primary Meige syndrome was diagnosed by an experienced neurologist, Ruen Liu. The exclusion criteria were as follows: 1. neurological diseases other than Meige syndrome and/or serious mental illness; 2. metabolic diseases, such as diabetes, hyperthyroidism or hypothyroidism; 3. positive urine toxicology or pregnancy test before any scan; 4. psychotropic medication use within the past 2 months; and 5. other serious systemic diseases, such as severe organic heart disease, severe lung, liver and kidney dysfunction, or coagulation dysfunction.

Written informed consent was obtained from each participant, and this study was approved by the institutional review board of Peking University People’s Hospital (2020PHB065-01). All methods were performed in accordance with relevant guidelines and regulations.

### PET Scanning

All patients fasted for at least 6 h before PET/CT imaging, and blood glucose levels were controlled to below 6.0 mmol/L. ^18^F-FDG (provided by Atom High-Tech Co., Ltd., Beijing, China) was injected intravenously at 5.55 MBq/kg (0.15 mCi/kg) (Zhou et al., 2020). After resting for 60 min in a dark, quiet environment, each patient lay in a supine position on the examination bed and then underwent head PET scans for 8 min. The spatial resolution of the scanner was 4.2-mm full width at half maximum (FWHM) in the axial, sagittal or coronal plane. The PET scan used the 3-dimensional (3D) mode. PET data were obtained using exactly the same conditions and imaging procedure (acquisition and image reconstruction) and the same PET scanner before and 6 months after surgery.

### Pre-processing of PET Data

PET images were analysed using SPM8^1^ in the MATLAB R2016a programming environment (The MathWorks, Natick, MA). PET data were pre-processed before analysis. First, spatial normalization was performed using the standard template of the Montreal Neurological Institute (MNI). Second, smoothing was performed by convolution using an isotropic Gaussian kernel with an 8-mm FWHM to increase the signal-to-noise ratio.

### Surgical Procedures and Deep Brain Stimulation Programming

The surgical procedure for bilateral STN-DBS was consistent with a previous study from our team (Ouyang et al., 2020). In brief, patients underwent bilateral stereotactic surgery under local anesthesia. The subthalamic nucleus was located by combining stereotactic MRI with microelectrode recording. The STN is located 2–3 mm behind the midpoint of the anteroposterior commissure, 12–14 mm lateral, and 4–6 mm below the plane of the anteroposterior commissure. A DBS electrode (model L302, PINS Medical, Beijing, China) and pulse generator (G102R, PINS Medical) were implanted, and the final position of the electrode was confirmed by neuroimaging. One month after surgery, stimulation was initiated. The optimal stimulation settings were progressively adjusted according to the patient’s response. The standard pulse setting was 60 μs in duration at 130 Hz, with the voltage adjusted to the individual patient. In addition, based on each patient’s response to neurostimulation, the parameters could be progressively adjusted at outpatient follow-up or by a telemedical application.

To detect changes in motor function, Burke–Fahn–Marsden Dystonia Rating Scale scores were obtained for the movement (BFMDRS-M) and disability (BFMDRS-D) subscales (Burke et al., 1985) (scores range from 0–40 and 0–30, respectively, with higher scores indicating greater impairment) based on evaluation of video recordings obtained at specific time points: 3 days before DBS (baseline) surgery and 6 months after surgery. The severity of dystonia in each patient was evaluated by an independent movement disorder neurologist (Hu Ding) who was not involved in the surgery or DBS programming.

### Statistical Analysis

Statistical analysis of differences in demographic characteristics between groups: Statistical significance between quantitative variables was assessed by X2 tests, with Yates’s or Fisher’s correction if necessary. Student’s *t*-tests were performed to evaluate data that followed a normal distribution. Bonferroni correction was applied for multiple comparisons. Significant differences between groups were identified at *P* < 0.05. Numerical variables are expressed as the mean ± SD. Qualitative variables are described as the absolute values of individuals in distinct groups. Statistical analyses were performed using SPSS 25.0 (IBM Corp., Armonk, NY, United States).

With the assistance of SPM8, the smoothed images were subjected to whole-brain voxelwise statistical comparison (independent two-sample *t*-test) before and 6 months after surgery. Then, we controlled for age, sex, and glucose variability. The output of the comparison was an SPM t-Map showing clusters of statistically significant voxels. Using xjView software (version 9.6^2^), MNI spatial localization and image display of brain regions with local differences in brain glucose metabolism were performed. Metabolism analysis was conducted using whole-brain voxelwise statistics to evaluate differences in whole-brain glucose metabolism between groups, and the statistical threshold was set as *P* < 0.001 [*P*-value after familywise error correction (PFWE) < 0.05, number of effective thresholds (K) = 50 voxels]. Only clusters with more than 50 voxels were considered statistically significant brain regions.

## Results

### Patients

A total of 25 Meige syndrome patients (11 males; 14 females) were included, and the age of the participants ranged from 33 to 77 years (53.60 ± 10.74). The disease duration in patients with Meige syndrome was 5.69 ± 5.82 years. The stimulation parameters were as follows: mean frequency = 136.60 ± 14.49 Hz, range = 130–185 Hz; mean pulse width = 64.40 ± 9.61 μs, range = 55–90 μs; and mean amplitude 2.78 ± 0.93 V, range = 1.23–4.70 V. The mean body mass index (BMI) was 25.28 ± 3.17 kg/m^2^ (Table 1).

**TABLE 1:** Baseline patient characteristics.

Characteristic*	Value
Age-yr	53.60 ± 10.74
Female sex-no. (%)	14 (56.00%)
Disease duration-yr	5.69 ± 5.82
Body Mass Index (BMI)-kg/m^2^	25.28 ± 3.17
Mean frequency- Hz	136.60 ± 14.49
Mean pulse width-μs	64.40 ± 9.61
Mean amplitude-V	2.78 ± 0.93

*Plus-minus values are means ±SD.

Compared to preoperative scores, there was a significant decrease in BFMDRS-M scores after 6 months of STN-DBS, from 10.02 ± 3.99 to 4.00 ± 2.69 (*p* < 0.001), with a mean improvement of 60.08% compared to preoperative scores. The BFMDRS-D scores also decreased significantly from 4.52 ± 2.90 to 0.64 ± 1.29 (*p* < 0.001), with a mean improvement of 85.84% compared to preoperative scores (Table 2).

**TABLE 2:** Burke–Fahn–Marsden dystonia rating scale scores at baseline and 6 months postoperatively.

Characteristic*	Baseline	6 months	*P*-value	Mean change at 6 months from baseline (95% CI)
BFMDRS-M (range, 0–40) §	10.02 ± 3.99	4.00 ± 2.69	<0.001	−6.02 (−7.24 to −4.80)
Eye (range, 0–8)	5.98 ± 2.06	2.42 ± 1.40	<0.001	−3.56 (−4.395 to −2.73)
Mouth (range, 0–8)	3.16 ± 2.52	1.38 ± 1.52	<0.001	−1.78 (−2.52 to −1.04)
Speech & Swallowing (range, 0–16)	0.56 ± 1.78	0.04 ± 0.20	0.142	−0.52 (−1.23 to 0.19)
Neck (range, 0–8)	0.32 ± 1.25	0.16 ± 0.55	<0.327	−0.16 (−0.49 to 0.17)
BFMDRS-D (range, 0–30) §	4.52 ± 2.90	0.64 ± 1.29	<0.001	−3.88 (−4.91 to −2.85)
Speech (range, 0–4)	0.36 ± 0.49	0.00 ± 0.00	0.001	−0.36 (−0.56 to −0.16)
Writing (range, 0–4)	1.28 ± 1.06	0.16 ± 0.37	<0.001	−1.12 (−1.55 to −0.69)
Feeding (range, 0–4)	0.84 ± 0.55	0.12 ± 0.33	<0.001	−0.72 (−0.94 to −0.50)
Eating and swallowing (range, 0–4)	0.04 ± 0.20	0.00 ± 0.00	0.327	−0.04 (−0.12 to 0.04)
Hygiene (range, 0–4)	0.32 ± 0.48	0.04 ± 0.20	0.005	−0.28 (−0.47 to −0.09)
Dressing (range, 0–6)	0.08 ± 0.28	0.00 ± 0.00	0.161	−0.08 (−0.19 to 0.03)
Walking (range, 0–4)	1.60 ± 1.08	0.32 ± 0.62	<0.001	−1.28 (−1.67 to −0.89)

*Plus-minus values are means ±SD.

### Glucose Hypermetabolism in Meige Syndrome

**FIGURE 1:**
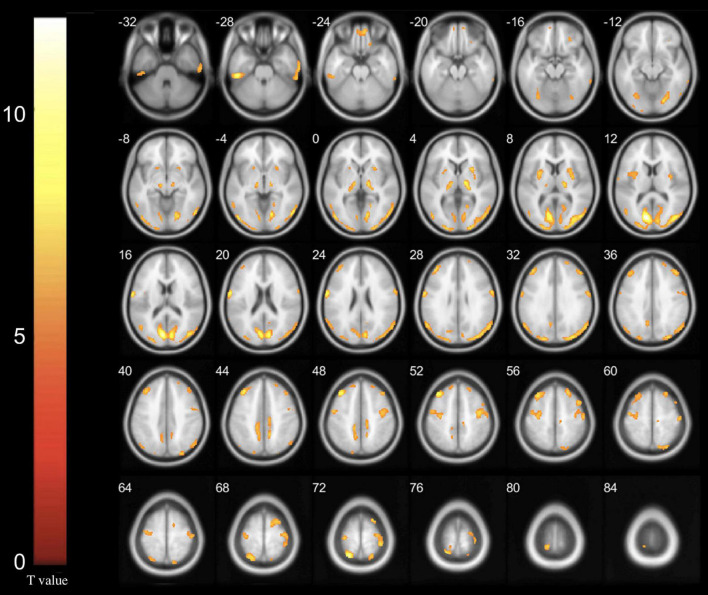
Increased glucose metabolism regions in Meige syndrome in 6 months after STN-DBS surgery (*P* < 0.001). In the left hemisphere, hypermetabolism was found in the occipital lobe, superior parietal gyrus, postcentral gyrus and thalamus. In the right hemisphere, hypermetabolism was found in the lentiform nucleus, precuneus and precentral gyrus. In addition, the bilateral inferior temporal gyrus and middle frontal gyrus exhibited glucose hypermetabolism. (Cluster size > 50 voxels. The color bar indicates the T-value).

After controlling for the effects of age, sex, and glucose level, multiple significant differences in the local brain glucose metabolism rate were revealed before and 6 months after surgery. We observed glucose hypermetabolism in the left hemisphere in the occipital lobe, superior parietal gyrus, postcentral gyrus and thalamus and in the right hemisphere in the lentiform nucleus, precuneus and precentral gyrus in patients with Meige syndrome receiving DBS. In addition, the bilateral inferior temporal gyrus and middle frontal gyrus were exhibited glucose hypermetabolism (Table 3 and Figure 1). No hypometabolic clusters were observed in this study.

**TABLE 3:** Regions with increased glucose metabolism in Meige syndrome in 6 months after STN-DBS surgery.

			Coordinates				
			
Brain region*	Side	K_E_	*x*	*y*	*z*	*T*-value	*Z*-value
Inferior temporal gyrus	Right	235	64	–20	–30	4.35	4.06
Inferior temporal gyrus	Left	202	–48	–30	–28	6.12	5.18
Middle frontal gyrus	Right	218	46	36	34	4.38	4.01
Middle frontal gyrus	Left	787	–36	26	50	5.54	4.80
Occipital lobe	Left	5319	–10	–80	12	7.31	5.88
Lentiform nucleus	Right	240	26	10	4	4.42	4.00
Precuneus	Right	214	12	–50	44	4.70	4.20
Precentral gyrus	Right	936	42	–10	50	4.55	4.09
Superior parietal gyrus	Left	343	–22	–60	72	6.21	5.23
Postcentral gyrus	Left	244	–62	–2	22	5.49	4.77
Thalamus	Left	195	–12	–16	2	4.36	4.03

*Plus-minus values are means ±SD.

## Discussion

Meige syndrome is a rare focal dystonia of the nervous system that was first reported by Henry Meige in 1910. This disease primarily causes blepharospasm, various forms of dystonia in the facial muscles and jaw and neck muscles and is more common in the elderly (Weiner et al., 1981). The etiology and pathogenesis of Meige syndrome have not been fully elucidated. Current studies of dystonia suggest that loss of inhibition at different levels in the central nervous system leads to hyperactivity in patients (Dresel et al., 2006). The spinal cord, brain stem, basal ganglia and cerebral cortex are dysfunctional, leading to the symptoms of dystonia (Prescott et al., 2013). In recent years, it has been an important research direction to explore biomarkers related to neurodegenerative diseases using imaging technology to determine the neuropathological mechanism corresponding to the clinical manifestations of diseases and to assist with clinical diagnosis and disease course monitoring. In our previous magnetic resonance imaging (MRI)-related study, we compared a Meige syndrome group to a healthy control group in a voxel-based morphology analysis. We observed decreased gray matter volume in the precuneus and believe it is involved in the development of Meige syndrome (Liu et al., 2020). However, we did not observe involvement of the basal ganglia or motor cortex in the pathophysiology of this disorder. As a traditional functional imaging method, ^18^F-fluoro-D-glucose (FDG)-PET can directly detect changes in glucose metabolism to reflect the activity of brain tissue, which is a special advantage over other imaging modes (Jueptner and Weiller, 1995). In our PET study, hypometabolism in the globus pallidus and thalamus was observed (Liu et al., 2021b). This may indicate basal ganglia-thalamocortical motor circuit abnormalities as a pathogenic mechanism of Meige syndrome.

The safety and efficacy of deep brain stimulation (DBS) have been gradually recognized, and it has become an important method for the treatment of Meige syndrome. Commonly used clinical targets include the globus pallidus internus (GPi) and subthalamic nucleus (STN), which have proven effective for the treatment of Meige syndrome by our research team (Hao et al., 2020; Ouyang et al., 2021). Comparing the effects of these two targets, we also found that STN-DBS and GPi-DBS exerted similar effects in Meige syndrome (Liu et al., 2021a). However, the mechanism of DBS treatment remains unclear. PET can be used not only to explore the pathogenesis of this disease but also to evaluate the effects and mechanism of DBS treatment (Eidelberg and Edwards, 2000). A PET-related study of Parkinson’s disease revealed that the reduction of symptoms after GPi-DBS was accompanied by a decrease in globus pallidus metabolism and an increase in premotor cortex metabolism (Fukuda et al., 2001). In this study, 25 patients with Meige syndrome who were treated with bilateral STN-DBS were examined by PET before and 6 months after surgery. We investigated the characteristics and mechanism of bilateral STN-DBS on cerebral local glucose metabolism in patients with Meige syndrome. We observed significant metabolic hypermetabolism primarily in the temporal gyrus, frontal gyrus, occipital lobe, lentiform nucleus, precuneus, precentral gyrus, superior parietal gyrus, postcentral gyrus and thalamus (Figure 1). This not only verified the results of our previous study but also verified basal ganglia-thalamocortical motor circuit abnormalities as a pathogenic mechanism of Meige syndrome, and this abnormal metabolic pattern is regulated by STN-DBS. In addition, the baseline characteristics and clinical efficacy of the patients in this group were similar to those of previous studies at our center, which was representative (Liu et al., 2020, 2021a; Ouyang et al., 2021). BFMDRS-M and BFMDRS-D scores were improved by 60.08 and 85.84%, respectively, confirming our previous findings that STN-DBS was effective for the treatment of Meige syndrome (Liu et al., 2021a).

The effect of STN-DBS on brain metabolism in patients with Meige syndrome has not yet been studied. Overall, metabolism in the brain increased in response to STN-DBS, and this change was consistent with improvement of clinical symptoms. The lentiform nucleus, precuneus, precentral gyrus, superior parietal gyrus, postcentral gyrus and thalamus, which have been shown to be associated with Meige syndrome in our previous studies (Liu et al., 2020, 2021b), were metabolically more active after STN-DBS. Patients with dystonia exhibit functional abnormalities at different levels of the cortex and subcortex, which are generally considered to be related to the basal ganglia-thalamocortical motor circuit (Colosimo et al., 2010; Neychev et al., 2011). Basal ganglia dysfunction is considered to be closely related to dystonia (Vidailhet et al., 2009). A previous study suggested that primary dystonia may be due to pathophysiological changes in the lentiform nucleus that lead to involuntary muscle contractions. A study by Becker et al. (1999) demonstrated that copper in the basal ganglia region was significantly increased in patients with primary dystonia, affecting cellular function and potentially leading to cell death by modulating enzyme activities or gene expression (Uauy et al., 1998). STN-DBS promotes the release of excitatory glutamate transmitters, increases nerve excitability and improves nerve function (Moldovan et al., 2015). In addition, the precuneus is widely associated with the auxiliary motor areas and the anterior cingulate gyrus. At the subcortical level, the precuneus is primarily associated with the claustrum and putamen and projects fibers to the brainstem (Leichnetz, 2001). Increased metabolism in the precuneus may ameliorate the abnormal cortical integration of Meige syndrome and thus improve the symptoms. At the cortical level, our study also showed that STN-DBS stimulates increased metabolism in the parietal and occipital lobes because the parietal occipital region integrates the peripheral higher somatosensory association area and can adjust motor function according to the state of the body (Cury et al., 2015). STN-DBS improves the above advanced central neuroregulatory functions, thus improving neurological function in patients with Meige syndrome. The frontal cortex is an important part of the emotional center pathway, and positive emotions lead to increased glucose metabolism in the frontal cortex (Cacioppo and Gardner, 1999). The temporal lobe plays an important role in visual and auditory integration and regulation, and it is closely related to the frontal lobe (Ono et al., 2014). Dystonia patients exhibit varying degrees of anxiety, depression and other emotional disorders but also display different degrees of visual and spatial dysfunction and other non-motor symptoms (Kuyper et al., 2011). Our previous study showed that STN-DBS was associated with superior improvements in depression and anxiety symptoms (Liu et al., 2021a). In this study, we suggest that STN-DBS may improve non-motor symptoms in patients with Meige syndrome by elevating metabolism in the frontal and temporal lobes. However, due to the short follow-up period (6 months), this study did not evaluate the postoperative non-motor symptoms of patients with Meige syndrome. Further studies are needed to evaluate the effects of DBS treatment on non-motor symptoms such as anxiety and depression and related brain metabolism changes. Finally, an fMRI study by Dresel et al. (2006) demonstrated a significant abnormal reduction in activation of the posterior central gyrus in patients with Meige syndrome, while no corresponding changes were observed in patients with blepharospasm. Therefore, this study suggested that the abnormal reduction in activation of the posterior central gyrus was characteristic of Meige syndrome and was closely related to its pathogenesis (Dresel et al., 2006). The “sensory trick” in Meige syndrome may be related to the deficiency of sensorimotor integration level in patients with dystonia, so we believe that this disorder of advanced sensory processing is also closely related to the appearance of dystonia motor symptoms (Lo et al., 2007). Therefore, increased metabolism in the anterior and posterior central gyrus may be the mechanism by which STN-DBS improves motor symptoms in patients with Meige syndrome.

The results of this study reflect the corrective effect of STN-DBS on abnormal basal ganglia-thalamocortical motor circuits in patients with Meige syndrome, which may be related to the excitatory effects on neuromodulation caused by regular local stimulation after stimulator implantation of the STN nucleus. At present, many Parkinson’s related studies have reported similar effects of elevated brain metabolism in response to STN-DBS. In some studies, PET scans of patients with Parkinson’s disease after STN-DBS were performed with electrical stimulation on and off, and symptomatic therapeutic electrical stimulation significantly increased the metabolism of the midbrain region around the electrodes (Hilker et al., 2004; Lewis et al., 2007). It has also been reported that STN-DBS may increase metabolism in the cingulate gyrus (Payoux et al., 2004; Haslinger et al., 2005). Our findings indicate that STN-DBS exerts a significant effect on metabolic level in the brain, which may be an important mechanism for the treatment of Meige syndrome using STN-DBS. In conclusion, by comparing PET scans of patients with Meige syndrome before and after STN-DBS treatment, this study found that STN-DBS stimulates the basal ganglia-thalamocortical motor circuit in patients with Meige syndrome, attenuating symptoms of dystonic disorder. The changes in brain metabolism, as the imaging representation of the regulation of this circuit, objectively reflect the above process.

However, in this study, metabolic changes in some brain regions were unilateral only, and such asymmetric changes have been reported similarly in other dystonia studies (Granert et al., 2011). However, there was no significant difference in bilateral clinical symptoms among the enrolled patients in this study, so the cause of unilateral metabolic changes could not be identified at this time, and further studies are needed to clarify this phenomenon. In addition, the correlation between the metabolic changes and behaviors in patients with Meige syndrome was not investigated. We plan to examine this in future research. In addition, although we used mask to control image spill over in the standardized step, the ROIs are not big enough to avoid risk of spill over due to the limit spatial resolution in this study. And the use of standard ROIs does not allow the correction for partial volume effects that may be pronounced in small structures. In future studies, we will collect MRI data and use a subject-specific atlas derived from individual structural T1-MRI to perform correction.

## Conclusion

Previous studies have proven that there is a pattern of abnormal metabolic regions and pathophysiological networks in Meige syndrome patients. In this study, by comparing the PET scans of patients with Meige syndrome before and 6 months after STN-DBS treatment, we found that metabolism was modified after STN-DBS. Our findings indicate that STN-DBS has a significant effect on metabolic level in the brain, which may be an important mechanism for the treatment of Meige syndrome using STN-DBS.

## Data Availability Statement

The raw data supporting the conclusions of this article will be made available by the authors, without undue reservation.

## Ethics Statement

The studies involving human participants were reviewed and approved by The Ethics Committee of Peking University People’s Hospital (2020PHB065-01). The patients/participants provided their written informed consent to participate in this study.

## Author Contributions

JL: literature search, study design, data collection, data interpretation, and writing. LL: literature search, data analysis, data interpretation, and writing. RL: study design and provided feedback on all manuscript texts. YL, QW, and HD: data collection and provided feedback on all manuscript texts. All authors contributed to the article and approved the submitted version.

## Conflict of Interest

The authors declare that the research was conducted in the absence of any commercial or financial relationships that could be construed as a potential conflict of interest.

## Publisher’s Note

All claims expressed in this article are solely those of the authors and do not necessarily represent those of their affiliated organizations, or those of the publisher, the editors and the reviewers. Any product that may be evaluated in this article, or claim that may be made by its manufacturer, is not guaranteed or endorsed by the publisher.

## Footnotes

1
https://www.fil.ion.ucl.ac.uk/spm

2
https://www.alivelearn.net/xjview/
